# How I do it – extracranial-intracranial bypass surgery integrating STA-MCA anastomosis, synangiosis, MMA supply, and preservation of recipient artery perforators: the Integrated Method

**DOI:** 10.1007/s00701-025-06689-z

**Published:** 2025-10-04

**Authors:** Milad Neyazi, Rajiv Kumar Khajuria, Katharina Faust, Sajjad Muhammad

**Affiliations:** Department of Neurosurgery, Medical Faculty and University Hospital Düsseldorf, Heinrich Heine University Düsseldorf, Moorenstr. 5, Düsseldorf, Germany

**Keywords:** Extracranial-intracranial bypass, STA-MCA anastomosis, Moyamoya disease, Encephaloduromyosynangiosis, Middle meningeal artery, Cerebral revascularization, Indirect bypass, Direct bypass, Neurovascular surgery, Perforator preservation

## Abstract

**Background:**

Extracranial-intracranial (EC-IC) bypass is an effective revascularization technique for flow augmentation in conditions like Moyamoya disease and ischemic cerebrovascular disorders. The approach described here combines direct bypass (STA-MCA anastomosis), indirect bypass (encephaloduromyosynangiosis, EDMS), preservation of the middle meningeal artery (MMA), and preservation of recipient artery perforators to optimize outcomes.

**Method:**

This article outlines a comprehensive surgical strategy emphasizing anatomical considerations, procedural nuances, and the integration of direct and indirect techniques while preserving MMA and recipient artery perforators to augment collateral circulation.

**Conclusion:**

The Integrated Method described here encompasses four technical aspects so as to enhance revascularization, minimize complications, and improve long-term outcome in carefully selected patients.

**Supplementary Information:**

The online version contains supplementary material available at 10.1007/s00701-025-06689-z.

## Introduction

Cerebrovascular revascularization strategies for ischemic conditions, particularly in Moyamoya disease, often face challenges in achieving sustained blood flow augmentation. Direct bypass, such as STA-MCA anastomosis, provides immediate perfusion, while indirect techniques like encephaloduromyosynangiosis rely on angiogenesis over time [3, 5]. Preservation of the middle meningeal artery and recipient M4 artery perforators during surgery offers an additional conduit for neovascularization, enhancing indirect revascularization outcomes. This "How I Do It" article demonstrates the integration of these techniques, addressing their synergistic benefits.

## Relevant surgical anatomy

The superficial temporal artery (STA) serves as a critical extracranial donor vessel, with its parietal branch most commonly employed for anastomosis to the M4 segment of the middle cerebral artery (MCA). The MCA acts as the primary intracranial recipient vessel, typically located on the cortical surface within sulci. Preserving M4 perforators meliorates direct bypass perfusion as well as indirect bypass formation. Additionally, the middle meningeal artery (MMA) supplies the dura mater and can serve as a scaffold for angiogenesis when preserved. The temporal muscle and dura mater also play essential roles in synangiosis by forming substrates for neovascularization.

## Description of the technique

### Preoperative planning

Preoperative preparation begins with high-resolution vascular imaging, such as digital subtraction angiography (DSA), computed tomography angiography (CTA), or magnetic resonance angiography (MRA), and perfusion scans to map the vascular anatomy, quantify reserve capacity, and assess collateral circulation. A Doppler ultrasound is used to identify the course of the STA branches. During anesthesia, normocapnia, normothermia, and stable hemodynamics are meticulously maintained to reduce intraoperative complications.

### Surgical steps

**Fig. 1:**
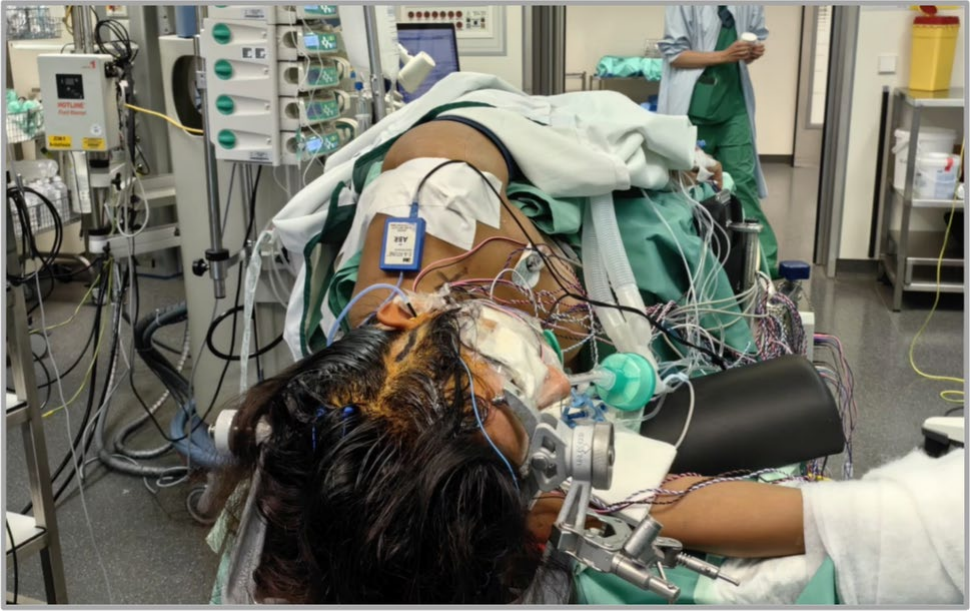
Patient positioning showing a 90° head tilt within the Mayfield clamp, with a linear temporal incision

**Fig. 2:**
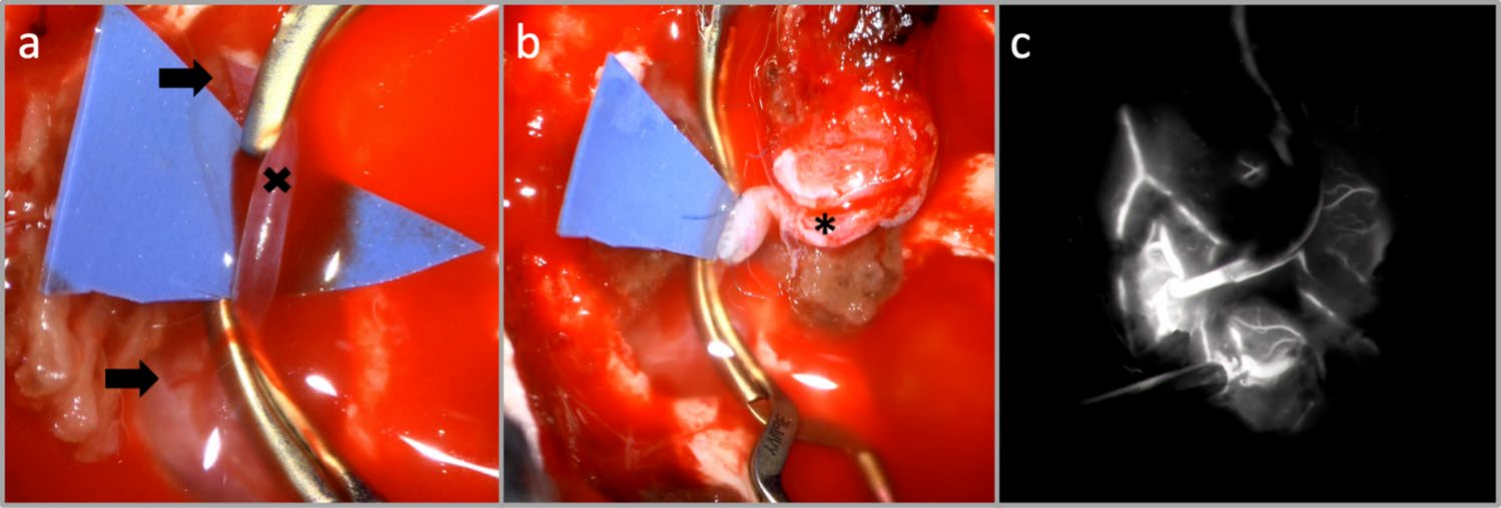
Intraoperative views demonstrating (**a**) the M4-recipient vessel (**x**) and the preservation of its perforators (→) (**b**) STA (*) – M4 anastomosis (**c**) confirmation of patency by intraoperative indocyanine green angiography

The patient’s head is rotated approx. 60° to the contralateral side and slightly extended. Intraoperative neurophysiological monitoring is employed (SSEPs, MEPs). Antiplatelet therapy is initiated 5 days preoperatively (acetylsalicylic acid 100 mg daily), and continued postoperatively. A linear incision is made along the STA’s parietal branch (Fig. 1). The STA is dissected along its course, trimmed obliquely to match the recipient vessel, and temporarily clipped before anastomosis. A Y-shaped incision is made through the temporal fascia. The muscle is split in a two-layer fashion and retracted anteriorly to expose the pterion. A tailored frontotemporal craniotomy (approx. 3 × 4 cm) is fashioned, centered over the Sylvian fissure. The dura is opened in a curvilinear fashion, extending approx. 2.5 to 3 cm to accommodate both direct and indirect bypass components. Concurrently, the MMA is preserved by avoiding unnecessary thermal or mechanical injury. The recipient MCA branch is identified, using indocyanine green fluorescence angiography, to evaluate perforators as well as its suitability for anastomosis. Once located, the MCA is prepared by dissecting the surrounding arachnoid mater. Special care is taken to preserve the recipient artery perforators. End-to-side STA-M4-anastomosis is then performed using 10–0 monofilament sutures. The patency of the anastomosis is confirmed intraoperatively with Doppler flowmetry and indocyanine green angiography (Fig. 2).

**Fig. 3:**
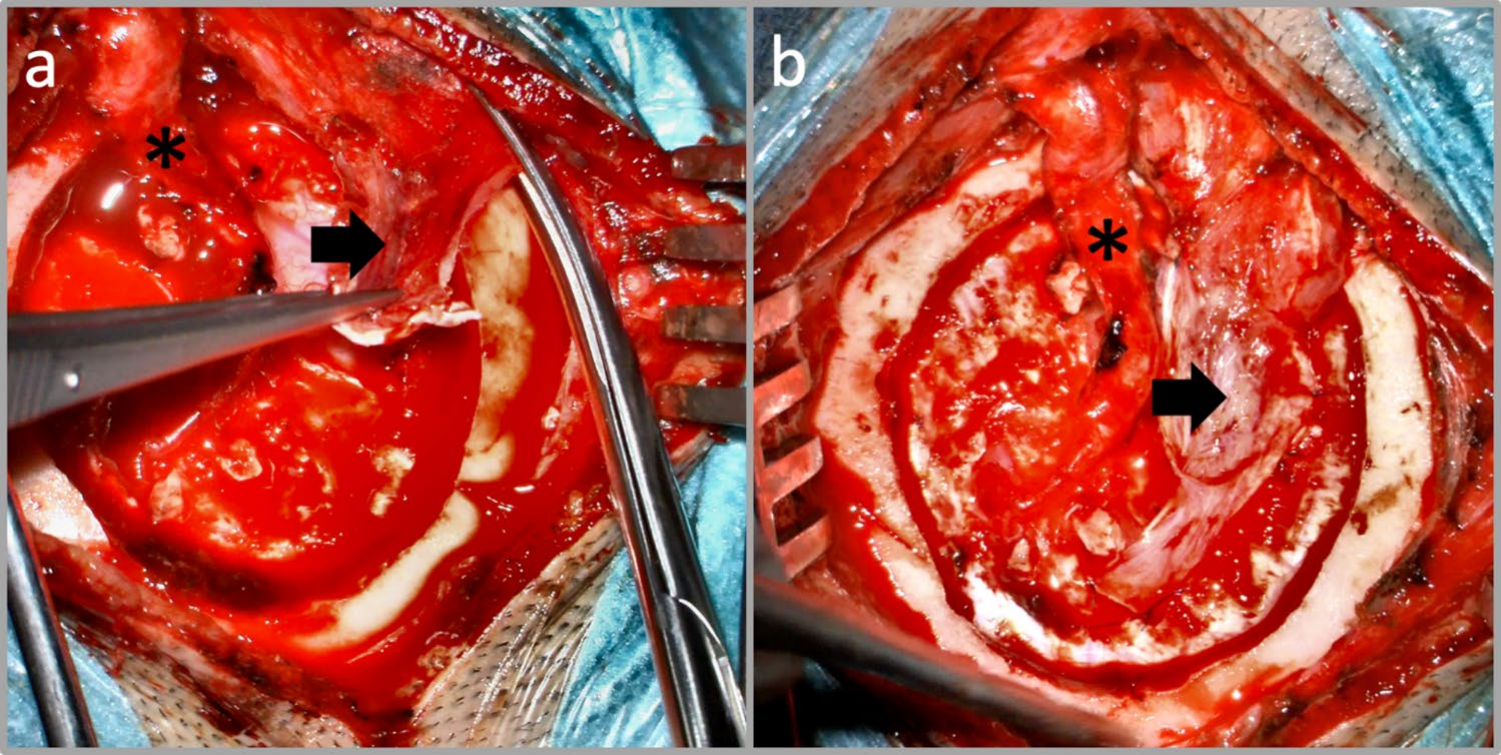
**a** Following direct bypass via STA (*) – M4 anastomosis, a temporal muscle flap (→) is prepared to place it directly over fenestral incisions within the dura mater to achieve encephaloduromyosynangiosis (**b**) final view prior to bone flap placement and closure

For indirect bypass, the temporal muscle is placed directly over the fenestral openings within the dura mater and sutured along its edges (Fig. 3) [4]. This arrangement ensures optimal contact for promoting angiogenesis. Additionally, the previously preserved MMA enables additional collateral vessel formation, augmenting the revascularization process.

**Fig. 4:**
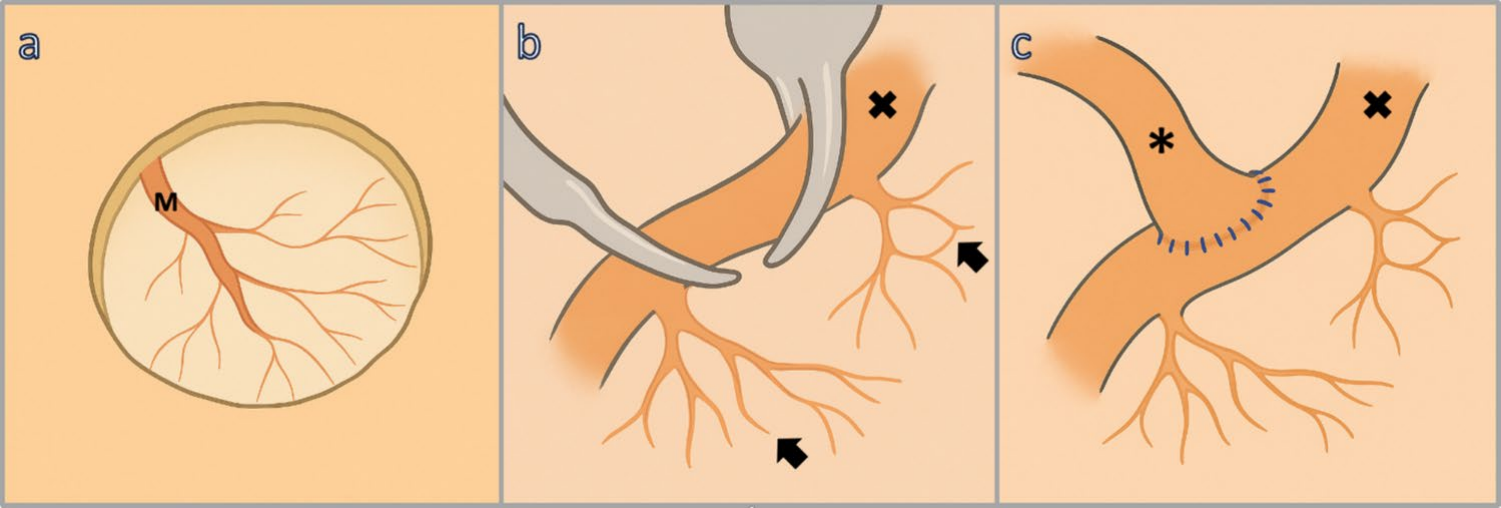
Schematic demonstration of key technical aspects: **a** sparing of the middle meningeal artery (M) during craniotomy and durotomy, **b** preservation of perforators (→) of recipient M4 (x), and **c** direct bypass via STA (*) – M4 (x) anastomosis

The closure involves replacing the partial bone flap and securing it with titanium plates. The scalp is closed in layers, ensuring adequate coverage and STA patency (Fig. 4).

## Indications

This integrated approach is indicated in both pediatric and adult patients with symptomatic Moyamoya disease, recurrent chronic ischemia with compromised reserve capacity in perfusion scans, inadequate collateral circulation as demonstrated on angiography, and progressive stroke unresponsive to medical therapy [2]. The procedure is performed as a first-line revascularization strategy; it is not intended as a secondary measure following indirect techniques [1].

## How to avoid complications

To avoid intraoperative ischemia, it is critical to maintain adequate perfusion pressures throughout the surgery. Anastomotic failure is prevented by ensuring meticulous microsurgical technique and intraoperative flow verification.

Potential postoperative complications include bypass occlusion, graft vasospasm, subdural hematoma, and wound healing issues at the STA donor site [7]. Hyperperfusion syndrome is managed through careful monitoring of neurological symptoms and controlled blood pressure reduction.

## Patient-specific information

Patients undergoing this procedure typically recover over 1–2 weeks, with gradual resolution of neurological deficits. Follow-up includes regular imaging to assess graft patency and angiogenesis. Outcomes generally demonstrate improved cerebral perfusion and reduced risk of ischemic or hemorrhagic events [6].

## Key points summary

1. STA-MCA anastomosis provides immediate revascularization.
2. Synangiosis promotes long-term collateral development.
3. MMA and perforator preservation enhances indirect bypass potential.
4. Integrating direct and indirect techniques synergizes outcomes.
5. Detailed preoperative planning ensures patient selection.
6. Meticulous surgical technique minimizes complications.
7. Postoperative care includes strict blood pressure management.
8. Imaging follow-up is crucial for assessing revascularization.
9. Patient education on expected recovery aids compliance.
10. This strategy is tailored for progressive ischemic conditions in pediatric and adult patients.

## Supplementary Information

Below is the link to the electronic supplementary material.

ESM 1 Supplementary Material l (MP4 420 MB)

## Data Availability

No datasets were generated or analysed during the current study.

## Abbreviations

EC-IC: Extracranial-Intracranial
STA: Superficial Temporal Artery
MCA: Middle Cerebral Artery
EDMS: Encephaloduromyosynangiosis
MMA: Middle Meningeal Artery
DSA: Digital Subtraction Angiography
CTA: Computed Tomography Angiography
MRA: Magnetic Resonance Angiography
ICG: Indocyanine Green
